# A case report on epicardial ultrasonography of coronary anastomoses using a stabilizing device without the use of ultrasound gel

**DOI:** 10.1186/s13019-019-0882-2

**Published:** 2019-03-13

**Authors:** Jan Jesper Andreasen, Dorte Nøhr, Alex Skovsbo Jørgensen

**Affiliations:** 1 Department of Cardiothoracic Surgery, Aalborg University Hospital, Hobrovej 18-22, 9000 Aalborg, Denmark; 2 Clinical Institute, Aalborg University, Sdr. Skovvej 15, 9000 Aalborg, Denmark; 3 Department of Health Science and Technology, Aalborg University, Fredrik Bajers Vej 7, 9220 Aalborg, Denmark

**Keywords:** Epicardial ultrasonography; coronary bypass surgery, Coronary anastomosis, Quality assessment

## Abstract

**Background:**

Intraoperative epicardial ultrasonography of coronary artery bypass graft anastomoses is a procedure used for anatomical quality assessment of peripheral anastomoses during coronary artery bypass grafting. However, it may be difficult to keep the ultrasound transducer in steady contact with the anastomoses on the beating heart without causing any deformation. Furthermore, we are not aware of any sterile ultrasound gel approved for application into the pericardial space.

**Case presentation:**

We report a method using a stabilizing connecting device for an ultrasound transducer to be used for visualization of coronary anastomoses without application of ultrasound gel during on-pump coronary bypass surgery.

**Conclusion:**

Use of a stabilizing device and coagulated blood from the patient as an alternative for ultrasound gel facilitates peroperative ultrasonography of coronary anastomoses. The procedure provides surgeons with non-deformed echocardiographic longitudinal and transverse images of all parts of the anastomoses.

**Trial registration:**

The patient participated in a still ongoing clinical feasibility study: Trial registration: ClinicalTrials.gov ID: NCT02919124; Registered September 29, 2016.

## Background

Intraoperative quality assessment of peripheral coronary anastomoses during coronary artery bypass grafting (CABG) has come into increased focus as it offers surgeons the opportunity to detect technical failures mandating revision of the bypass graft before the chest closure. Failures that may be detected are e.g. twisting of the graft, anastomosis stenosis, a narrow toe of the anastomosis, kinked graft etc. [1, 2].

Transit-time flow measurement (TTFM) during CABG is well described and has been used for approximately two decades for functional graft assessment [3, 4]. Recently, use of high-frequency intraoperative epicardial ultrasonography of coronary anastomoses has been shown to be complementary to TTFM improving the diagnostic accuracy of intraoperative graft assessment [5]. Optimal acoustic and stable contact between the ultrasound probe and the anastomoses to be examined are needed without the risk of distortion of the anastomoses.

We are not aware of any ultrasound gel approved for application into the pericardial space during cardiac surgery in humans. The objective of this case report is to describe a method for ultrasound imaging of coronary artery anastomoses during on-pump CABG using the patient’s coagulated blood instead of ultrasound gel together with a stabilizing device.

## Case presentation

A male, 58 years of age, was referred for an elective CABG procedure due to stable angina pectoris. Five weeks earlier, he suffered a ST-elevation acute myocardial infarction due to three-vessel coronary artery disease. Coronary angiography showed an occluded right coronary artery (RCA) and significant stenoses located to the obtuse marginal branch (OM) and the left anterior descending coronary artery (LAD). The occluded RCA was treated with emergent percutaneous balloon angioplasty and stenting within 6 h after the myocardial infarction developed. The patient experienced a temporary 3^o^-atrio-ventricular block, but was discharged in sinus rhythm 4 days later after an otherwise uneventful course. Following a heart team discussion, it was decided to perform surgical revascularisation of the left sided coronary arteries 4 weeks later. The left ventricular ejection fraction was 50%, and no valvular diseases were diagnosed by transthoracic echocardiography. Logistic EuroSCORE II was 0.79%. After surgery, the patient was discharged following an uneventful course.

### Surgical procedure

Surgery was performed on-pump. Routine thoracic surgical and anaesthetic procedures were employed, and transoesophageal echocardiography was available. A saphenous vein graft was harvested endoscopically and anastomosed end-to-side to the OM with the proximal anastomosis on the ascending aorta. A left internal mammary artery (LIMA) pedicel graft was anastomosed to the LAD. Cold blood cardioplegia was administered twice in the aortic root during 29 min of aortic cross-clamping. Perfusion time was 70 min.

### High-resolution epicardial ultrasonography

**Fig. 1:**
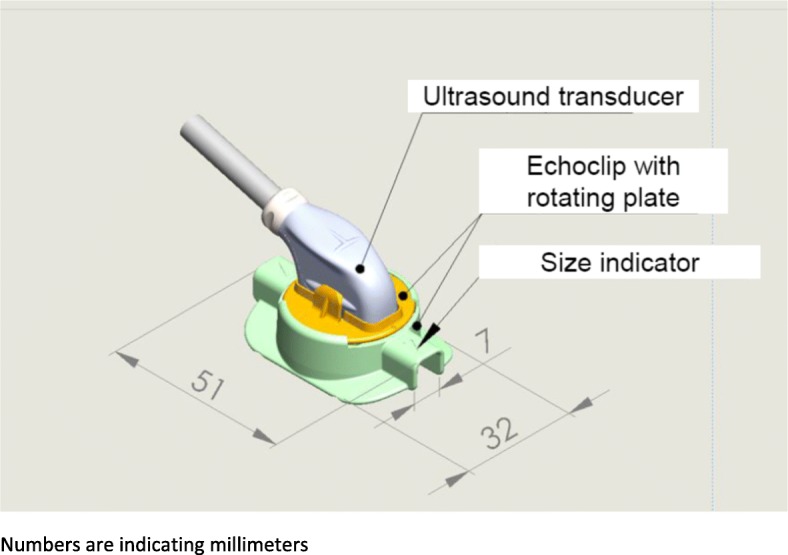
Drawing of the echoclip stabilizing device

The Medistim VeriQ™ System with a 15 MHz ultrasonography probe was used (Medistim A/S, Oslo, Norway). The ultrasonography probe was connected to a disposable stabilizing device (Echoclip, Aalborg University Hospital, Denmark) (Fig. 1). This device allows the anastomosis to be imaged without applying pressure on the graft when stabilising the heart with the stabiliser. The surgeon should make sure that the graft is located in the cavity of the stabilizer in order to avoid obstruction of the graft flow during the scanning procedure. The echoclip device is created such that slippery excess coagulated blood and ultrasound gel (if this will be approved in the future) are allowed to escape from the cavity during scanning. The echoclip device, which is not commercially available yet, is a disposable article, which comes in different sizes.

**Fig. 2:**
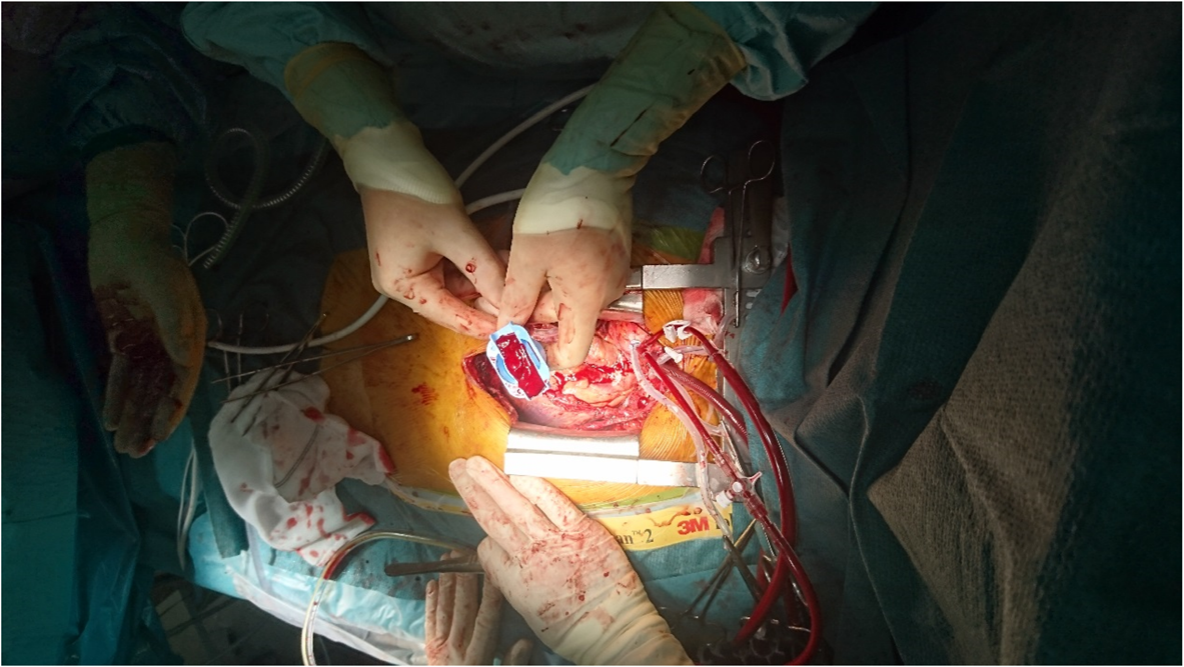
Coagulated blood placed in the cavity of the stabilizing device designed to receive the graft at the site of the coronary anastomosis

**Fig. 3:**
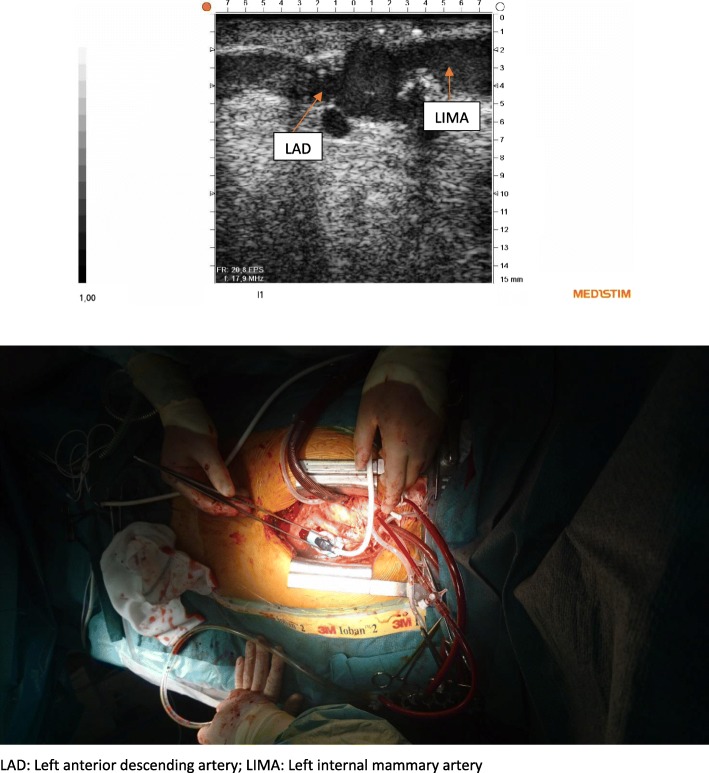
Epicardial ultrasonography of the left internal mammary artery anastomosis to the left anterior descending coronary artery in the longitudinal plane

**Fig. 4:**
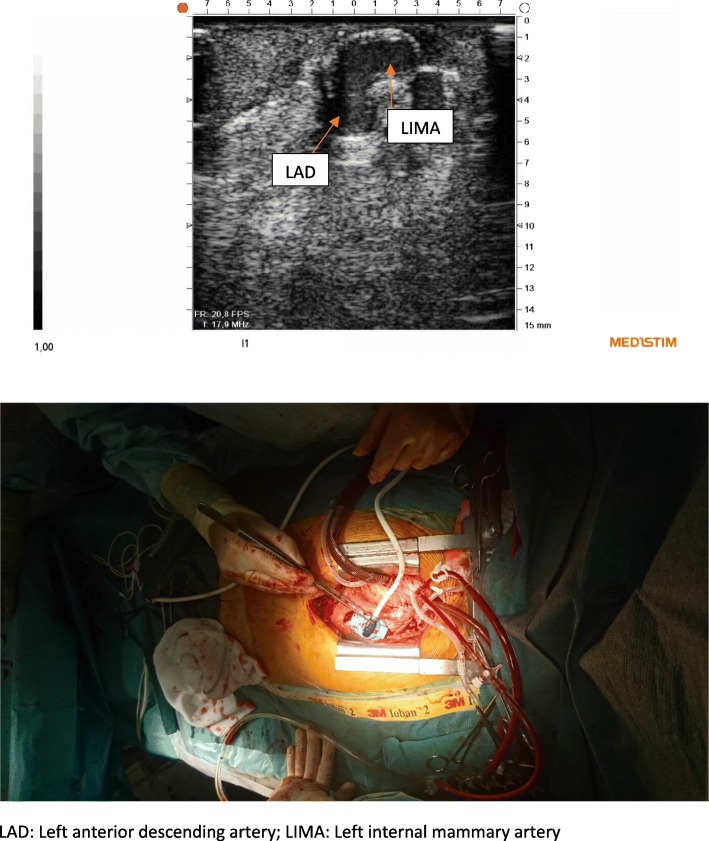
Epicardial ultrasonography of the left internal mammary artery anastomosis in the transverse plane

Instead of gel we used coagulated blood obtained from the patient before heparinization in order to obtain acoustic contact with the anastomosis. Coagulated blood was placed in the cavity of the stabilizer designed to receive a part of the graft and the anastomosis to be examined (Fig. 2). The peripheral vein graft anastomosis was validated during cross-clamp, while infusing cold blood cardioplegia directly into the vein graft. A rotating plate to which the imaging probe was secured allowed imaging of the coronary anastomosis both the longitudinal (Fig. 3) and transverse plane (Fig. 4). The LIMA anastomosis was validated after the cross-clamp was released while still on pump. Less than 5 min was used to obtain images of the heel, the central portion and the toe of the anastomoses in both the longitudinal and transverse planes. The coagulated blood was sucked away from the pericardial space after the imaging procedure. TTFM was performed in addition to epicardial ultrasonography.

## Discussion and conclusions

A combination of TTFM and high-frequency epicardial ultrasonography of peripheral bypass anastomoses facilitate quality assessment of coronary anastomoses offering a possibility for graft revision before closure of the chest [5]. However, surgeons have to overcome a learning curve especially in relation to the use of epicardial ultrasonography of peripheral coronary anastomoses. Experienced surgeons have indicated that the basics can be learned in about 10–20 grafts [1, 2].

We have described a reproducible and simple procedure to perform epicardial ultrasonography of peripheral coronary bypass anastomoses with use of a stabilizing device and with the use of the patients coagulated blood instead of ultrasound gel. Thus, the patients coagulated blood may be used until sterile ultrasound gel is approved for use in the pericardial space during cardiac surgery. It is a limitation, that this procedure can only be performed if coagulated blood is collected prior to heparinization. It remains to be proven, if coagulated blood can be used instead of gel without the use of a stabilizing device both during on-and off-pump cases and whether the procedure can be used during off-pump surgery. Development of a handle attached to the echoclip device will probably be needed if ultrasound imaging is to be performed during off-pump surgery on the backside of the heart, and if imaging is need after administration of protamine.

In conclusion, use of a stabilizing device and coagulated blood from the patient as an alternative for ultrasound gel facilitates peroperative ultrasonography of coronary anastomoses. The procedure provides surgeons with non-deformed echocardiographic longitudinal and transverse images of all parts of the anastomoses.

## Abbreviations

CABG: Coronary artery bypass grafting
LAD: Left anterior descending coronary artery
LIMA: Left internal mammary artery
OM: Obtuse marginal branch
RCA: Right coronary artery
TTFM: Transit-time flow measurement
